# Genomic data uncover complex hybridization and evolutionary history of the bunchberry species complex (*Cornus* L., Cornaceae)

**DOI:** 10.1093/hr/uhaf026

**Published:** 2025-01-22

**Authors:** Yanxia Sun, Wenbin Zhou, Qiu-Yun (Jenny) Xiang

**Affiliations:** State Key Laboratory of Plant Diversity and Specialty Crops, Wuhan Botanical Garden, Chinese Academy of Sciences, Wuhan 430074, China; Department of Plant and Microbial Biology, North Carolina State University, Raleigh, NC 27695, USA; Department of Biology, University of North Carolina, Chapel Hill, NC 27599, USA; Department of Plant and Microbial Biology, North Carolina State University, Raleigh, NC 27695, USA

Dear Editor,

Hybridization is an important force in plant evolution and speciation, and a powerful traditional method for target breeding of horticultural crops [1]. Investigating the occurrence, extent, and genetic consequences of natural hybridization in a group is not only critical to understanding the processes shaping plant diversity but also important to genetic breeding of ornamental plants by guiding the selection of wild relatives for hybridization. However, our knowledge on the dynamics of natural hybridization and its evolutionary outcomes in many plants, especially those restricted to special habitats and with horticultural importance/potentials, is highly limited.

The dwarf dogwoods, the *Arctocrania* clade of *Cornus* L. (Cornaceae), are four rhizomatous perennial species, *Cornus canadensis* L. (*Cc*), *C. suecica* L. (*Cs*), *C. unalaschkensis* Ledeb. (*Cu*), and *C. wardiana* Rushforth & Wahlsteen (*Cw*) (Fig. 1A), well known for their strong tolerance to low temperature and uses as ornamental and food plants. These plants are durable, attractive ground covers widely grown in hanging baskets or gardens, and make edible red fruits that are used for jams, jellies and fruit preserves. The boundaries of the four known species are blurred due to prevalent intermediate forms between *Cc* and *Cs* throughout the North American boreal–arctic distribution range of the clade, which are classified as *Cu*, *Cc* > *Cs* (morphologically more similar to *Cc*), and *Cs* > *Cc* (morphologically more similar to *Cs*) (Fig. S1) [2]. These three intermediate forms and the two morphological extremes represented by *Cc* and *Cs* comprise the bunchberry species complex (Table S1). The morphological variation suggested that hybridization may have been an important process driving the evolution of the group, leading to species formation and diversification and, maybe, also breakdown of species boundaries. To test the hypothesis, we conducted a study of the species complex by including 374 dwarf dogwood individuals from 88 populations across the distributional range covering morphological variation representing the known species and intermediate forms using genome-wide genetic polymorphisms at 43,137 variant sites from 1,126 ddRAD (double-digest restriction site-associated DNA) loci (Table S2). We integrated population genomics, phylogeography, and distribution modeling (Fig. S2; Tables S3 and S4) to document hybridization and gain insights into its role in shaping the evolution of the species complex and how climate changes have affected hybridization.

**Figure 1:**
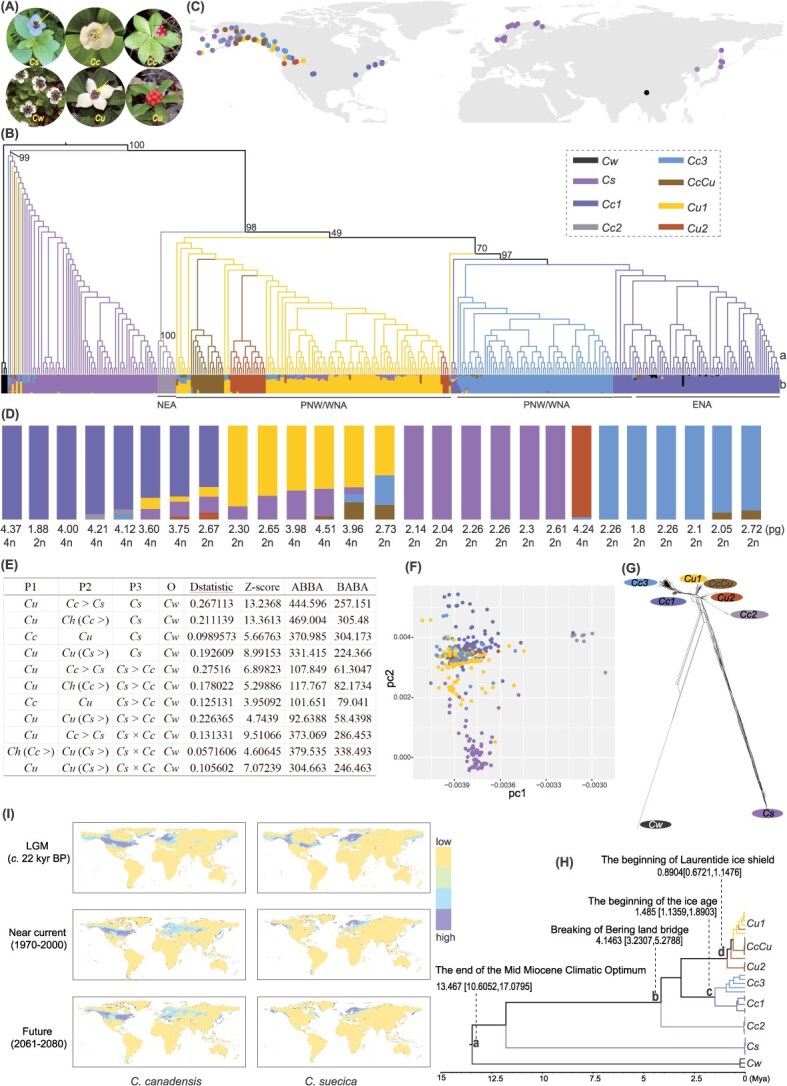
Population genomic analyses of the dwarf dogwoods. (A) Images of *Cornus canadensis*, *C. suecica*, *C. unalaschkensis*, and *C. wardiana* showing differences in petal color and leaf texture and morphology*.* The petals of the bunchberry complex are indicated with arrows. *Cc*, *C. canadensis*; *Cs*, *C. suecica*; *Cu*, *C. unalaschkensis*; *Cw*, *C. wardiana.* Image of *Cw* was cropped from a photo provided by Hugh Mc Allister. (B) Evolutionary relationships of the 374 dwarf dogwood individuals with genetic cluster memberships as inferred by ADMIXTURE under optimal *K* = 8. (a) ML (maximum likelihood) tree inferred from phylogenetic analysis using IQ-TREE, rooted using *Cw* based on the results from [6]. Bootstrap support values for main clades identified by the analysis are labeled. Colors of branches are consistent with the genetic cluster memberships identified by ADMIXTURE. (b) Plotted genetic components of all individuals identified by ADMIXTURE. General geographical distributions of *Cc* and *Cu* lineages are indicated: NEA, northeastern Asia; PNW, Pacific Northwest; ENA, eastern North America; WNA, western North America. (C) Geographical distribution of the 88 dwarf dogwood populations, with their genetic cluster membership inferred by ADMIXTURE shown in respective colors. (D) Genome size (2C) of 27 dwarf dogwood individuals estimated using flow cytometry. Putative ploidy levels of the 27 individuals inferred based on genome size with known ploidy published in [7] are also labeled. The genetic components of these individuals inferred by ADMIXTURE are indicated by color consistent with that in (B). (E) Results of the ABBA-BABA analysis. The analysis is based on the phylogeny of (((P1, P2), P3), O) as revealed in Fig. 1B, G and H and tests gene flow between P3 and P2 (*D* ≠ 0; *Z* > 3) and P3 and P1 (*D* ≠ 0; *Z* > 3). The samples of P1, P2, and P3 were assigned according to the taxon names labeled for each individual in Table D1 as identified based on morphology and the phylogeny revealed in Fig. 1B. O is the outgroup. BABA, number of SNPs shared between P1 and P3; ABBA, number of SNPs shared between P2 and P3; the analysis supports gene flow between P2 and P3. *Cc*, *C. canadensis*; *Cs*, *C. suecica*; *Cu*, *C. unalaschkensis*; *Cw*, *C. wardiana*; *Ch*, *C. hybrid* in Table S2*.* (F) Plots of two principal components showing genetic relationships among the 370 bunchberry species complex individuals. Colors represent their respective cluster membership inferred by ADMIXTURE. (G) Phylogenetic neighbor networks of the dwarf dogwoods computed with SplitsTree 4. The genetic cluster memberships of lineages are indicated in color consistent with (B). Parallel edges represent the splits computed from the data. (H) Estimation of divergence time using individuals with ‘pure’ ancestry from each genetic group identified by ADMIXTURE. Divergence times [mean and 95% CI in million years ago (Mya)] between main clades are labeled and annotated. Colors of branches correspond to respective taxa in other panels of the figure. (I) Estimated habitat suitability for *C. canadensis* and *C. suecica* during the last glacial maximum (LGM), the near current (1970–2000), and in the future (2061–2080) by MaxEnt based on available distribution locations of the species and the standard 19 WorldClim Bioclimatic variables.

Our analyses revealed eight ancestries for the 374 individuals: *Cw*, *Cs*, *Cc1*, *Cc2*, *Cc3*, *CcCu*, *Cu1*, and *Cu2* (Figs 1B, S3, S4, and S5; Table S2), each containing a portion of individuals with admixed ancestries, except *Cc2* from northeastern Asia (NEA) (Figs 1B, 1C, S4, and S5). The spatial pattern of genetic clusters and their phylogenetic relationships indicates a number of points: (i) hybridization and introgression within the species complex are prevalent and complicated (Figs 1B, S4, and S5), which was also supported by the *D*-statistic (Fig. 1E); (ii) *Cw*, *Cs*, and *Cc2* are geographically and genetically well separated (Figs. 1B, 1C); (iii) *Cc* and *Cu* are genetically and evolutionarily more diverse than previously known, showing divergence associated with geographic regions (Fig. 1B and C); (iv) *Cc* > *Cs* and *Cs* > *Cc* are not recognized as distinct genetic clusters but spread in other clusters (Fig. S4; Table S2); (v) the Pacific Northwest (PNW) region is a center of genetic diversity and heterogeneous hybridization (Figs 1B and S4); (vi) possible ancient hybridization between *Cw* and *Cc1* based on individuals showing significant admixture of ancestries of *Cw* and *Cc1* (Figs 1B, S4, and S5). In short, the patterns of genetic admixture combined with results from the *D*-statistic suggest the presence of hybrids at different generations, from *F1* to later backcross generations, from both ancient and more recent events in the clade, based on levels of introgression/admixture of genetic ancestries of different species.

Although *Cu* has been recognized as an allopolyploid species from hybridization between *Cs* and *Cc* [2], the genetic admixture and phylogeny suggest *Cu* was probably derived independently of *Cs*, but evolving within *Cc* during the Pleistocene (Fig. 1B and H). This timing of *Cu*’s origin is consistent with the previous hypothesis [2]. Limited data for flow cytometry showed a mixture of ploidy of the individuals in *Cu1* including those with admixed ancestries (Fig. 1D). The detection of the diploid *Cs* × *Cu1* genome points to a diploid ancestry of *Cu1* and suggests likely autotetraploidy in the *Cu1* group (Fig. 1D). Additional flow cytometry data from plants not included in the genetic analysis show a complex variation of genome size at a given location (Fig. S6; Table S5), suggesting dynamic changes in genome sizes that likely involved hybridization, aneuploidy, and/or polyploidy within and between taxa.

Divergence time estimation of the eight genetic groups shows a good match to several past climatic events (Fig. 1H), suggesting significant impacts of historical climate changes on the diversification and hybridization of the dwarf dogwoods. The successive early divergence of *Cw* and *Cs* was concordant with the ending of the Mid-Miocene Climatic Optimum. Given the detection of putative hybrids between *Cw* and *Cc1* in eastern North America (ENA) (Fig. S4), the divergence of *Cw* from *Cs + Cc-Cu* likely occurred in the north, where their ranges were once overlapping to facilitate hybridization, before it dispersed southward into the Sino-Himalayan region. The divergence between NEA *Cc2* and North America (NA) *Cc*-*Cu* corresponds to the breaking of the Bering land bridge [3], and the diversification of the NA lineage into the ENA *Cc1* and western North America (WNA) *Cc3*, *Cu1, Cu2*, and *CcCu* occurred during the mid-Pleistocene ice age when the grassland in central NA had well-developed isolating forests in the eastern and western USA and glaciation cycles significantly impacted the PNW region, where it is known to have provided refugia within refugia for organisms [4]. Hence, diversification of the *CcCu*-*Cu* subclade following the beginning of the Laurentide ice shield was likely promoted by combined effects of geographic isolation, remixing, hybridization, and polyploidization in refugia of the PNW during the glaciation periods.

Considering genetic differentiation, phylogenies, morphology, and distribution, we propose to recognize NEA *Cc2* as a new species with the name *Cornus orientalis* Y.X.Sun, W.B.Zhou & Q.-Y.Xiang, and two cryptic subspecies within *Cu* [*C. unalaschkensis* subsp. *unalaschkensis* (*Cu1* + *CcCu*) and *C. unalaschkensis* subsp. *borealis* (*Cu2*)], and two cryptic subspecies within *Cc* [*C. canadensis* subsp. *canadensis* (*Cc1*) and *C. canadensis* subsp. *pristina* (*Cc3*)]. Incomplete lineage sorting and gene flow are expected between species and subspecies, which explains why *CcCu* and *Cu2* individuals nested within *Cu1*.

It must be noted that the previously diverged lineages can face the risk of reverting to a single gene pool due to continuous hybridization and introgression in the PNW region (Fig. 1F). This concern is coupled with the predicted reduction of suitable habitats of *Cs* in the region (Fig. 1I). The modern suitable range of the species has significantly narrowed and shifted southward in Beringia, Alaska, western Canada, and NE America, compared with the last glacial maximum (LGM) (Fig. 1I). The future range of *Cs* is predicted to be highly reduced in the PNW, with loss in some parts of ENA. Shrinkage of the coastal arctic and alpine habitats due to sea level increase under climate warming will enhance the threat to *Cs* from hybridization with *Cc* and *Cu*.

In summary, our study based on genome-wide polymorphisms revealed hidden genetic structures of the bunchberry species complex. The evolutionary histories may have involved complicated hybridization, introgression, and polyploidization. Results from the present study support important roles of hybridization and climate changes in the diversification of species adapted to cold and subarctic regions that are facing the most threat of climatic warming [5]. The new knowledge learned from the study that all species are capable of hybridization presents opportunities to breed varieties with desired traits from different species or hybrid forms, such as *Cw*-like evergreen plants with white petals via artificial hybridization.

## Data Availability

The raw DNA sequencing reads of the 374 dwarf dogwoods have been deposited at the SRA repository under bioproject number PRJNA1167660. The supplementary data and methods generated in this study are available from the Dryad Digital Repository: http://datadryad.org/stash/share/7xnlhK-4MR0v34GlIBQFBDZ1uOymEJDInz7JsRyatQw. Additional data (e.g., SNP data matrices) related to this research are available from the first author.
