# Correction to: Genomic data uncover complex hybridization and evolutionary history of the bunchberry species complex (*Cornus* L., Cornaceae)

**DOI:** 10.1093/hr/uhaf161

**Published:** 2025-06-26

This is a correction to: Yanxia Sun, Wenbin Zhou, Qiu-Yun (Jenny) Xiang, Genomic data uncover complex hybridization and evolutionary history of the bunchberry species complex (*Cornus* L., Cornaceae), *Horticulture Research*, Volume 12, Issue 5, May 2025, https://doi.org/10.1093/hr/uhaf026

The following change has been made to the originally published manuscript. The name of the second co-author has been emended to read: “Wenbin Zhou”.

